# Age and Growth of the Round Stingray *Urotrygon rogersi*, a Particularly Fast-Growing and Short-Lived Elasmobranch

**DOI:** 10.1371/journal.pone.0096077

**Published:** 2014-04-28

**Authors:** Paola A. Mejía-Falla, Enric Cortés, Andrés F. Navia, Fernando A. Zapata

**Affiliations:** 1 Fundación Colombiana para la Investigación y Conservación de Tiburones y Rayas, SQUALUS, Carrera 60A No 11-39, Cali, Colombia; 2 Grupo de Investigación en Ecología de Arrecifes Coralinos, Departamento de Biología, Universidad del Valle. A.A. 25360, Cali, Colombia; 3 NOAA/National Marine Fisheries Service, Panama City, Florida, United States of America; University of California Davis, United States of America

## Abstract

We examined the age and growth of *Urotrygon rogersi* on the Colombian coast of the Eastern Tropical Pacific Ocean by directly estimating age using vertebral centra. We verified annual deposition of growth increments with marginal increment analysis. Eight growth curves were fitted to four data sets defined on the basis of the reproductive cycle (unadjusted or adjusted for age at first band) and size variables (disc width or total length). Model performance was evaluated using Akaike's Information Criterion (AIC), AIC weights and multi-model inference criteria. A two-phase growth function with adjusted age provided the best description of growth for females (based on five parameters, *DW_∞_*  =  20.1 cm, *k*  =  0.22 yr^–1^) and males (based on four and five parameters, *DW_∞_*  =  15.5 cm, *k*  =  0.65 yr^–1^). Median maturity of female and male *U. rogersi* is reached very fast (mean ± SE  =  1.0 ± 0.1 year). This is the first age and growth study for a species of the genus *Urotrygon* and results indicate that *U. rogersi* attains a smaller maximum size and has a shorter lifespan and lower median age at maturity than species of closely related genera. These life history traits are in contrast with those typically reported for other elasmobranchs.

## Introduction

Knowledge of age and growth characteristics allows construction of age-based population models and, together with the consideration of other life history aspects and removal rates by fisheries, can eventually lead to an assessment of the population status of a given species [1]. While target species have often been intensely studied, bycatch species are often ignored. These commercially unimportant species, such as the stingrays in the Family Urotrygonidae, are also impacted by fisheries and information on their life history is needed as input to formulate fisheries management decisions.

The round stingray *Urotrygon rogersi* (Jordan and Starks 1895) is an endemic batoid of the Eastern Tropical Pacific Ocean that occurs on soft bottoms in coastal and shallow zones at depths of 2 to 30 m [2]. It is the most abundant elasmobranch species in the bycatch of artisanal and industrial prawn trawl fisheries on the Colombian Pacific coast and does not have any commercial value [3].

This species is a specialist that feeds mainly on crustaceans and polychaetes, showing a strong diet overlap between sexes and size classes [4]. This aplacental viviparous species attains a maximum size of 20 cm disc width (DW), its median size at maturity is 11.5–11.8 cm DW in males and 11.8–12.3 cm DW in females, size at birth is 7.5–8.2 cm DW and 11.5–14.7 cm total length, gestation lasts about 5–6 months, and the reproductive cycle is triannual and aseasonal [5].

Seasonally reproducing species usually have relatively well-defined birth dates [6] and therefore the age of individuals can be determined with reasonable accuracy [7]. In contrast, non-seasonally reproducing species can have several reproductive peaks or reproduce throughout the year [6], and consequently the age at which the first growth band is formed in a vertebra is unknown. Therefore, age determination based on vertebral growth bands in these species requires that the data be adjusted, specifically by averaging the time between births and the formation of the first growth band [7].

Important considerations when fitting growth curves to observed size-at-age data include the metric of body size and the type of growth curve used. Most studies use total length, yet other metrics may be more relevant for some shark and batoid species [8]–[10]. Although the von Bertalanffy growth curve has been most extensively used to describe growth in fishes, use of more than one growth function to adequately characterize the growth of a given species has been recommended [11] and used in different elasmobranch species [10], [12]–[15]. However, few elasmobranch studies [13], [15], [16] have used multi-model inference, as proposed by Katsanevakis [17], to determine a model-averaged set of parameters across competing models incorporating uncertainty in model selection.

The objective of the present study was to use a multi-model approach to estimate age and growth parameters for *U. rogersi* from the coast of Colombia in the Eastern Tropical Pacific Ocean. To that end we considered four data sets based on: 1) two body size metrics (disc width and total length) as suggested by Cailliet et al. [11] for batoids; and 2) inclusion or not of the reproductive cycle of *U. rogersi*, specifically the time between births and month of first band formation. For each data set we compared eight growth models with varying number of parameters and used multi-model inference when necessary. Finally, median age at maturity of female and male *U. rogersi* was estimated from the best-fit growth curve.

## Materials and Methods

### Sample collection and study area

*Urotrygon rogersi* specimens were collected between 2006 and 2009 from the bycatch of the artisanal prawn trawl fishery in four locations of the Pacific coast of Colombia between 3° 56′ N - 77° 25′ W and 3° 00′ N - 77° 10′ W. This area is characterized by shallow waters (≤ 10 m depth) and sandy and muddy bottoms. Sex and maturity stage were recorded and disc width (DW, cm) and total length (TL, cm) measured in a straight line for each specimen. The stage of maturity (0-immature, 1-mature) was assigned from measurement and macroscopic examination of reproductive tracts following Mejía-Falla et al. [5]. Procedures and protocols used for sample collection were approved by the Universidad del Valle. Specimens were obtained dead from fishermen and fully assessed while live animals were only measured, sexed and returned to the sea.

### Treatment of vertebrae and reading of bands

The abdominal region of the vertebral column of each specimen was cut out and stored frozen. Vertebrae were manually cleaned by removing the connective tissue, washed with distilled water and allowed to dry. Each vertebra was fixed to a clear glass slide with resin (Crystalbond 509 or thermoplastic cement, Electron Microscopy Sciences, Hatfield, PA, USA) and sagittally sectioned with a Buehler 82 Isomet low-speed saw (Buehler, Lake Bluff, IL, USA) resulting in a “bow tie” section [18], [19]. After trials with different thicknesses and stains we found that an unstained 0.4 mm section produced the best result [20]. Each bow-tie section was observed in a stereomicroscope, and slightly polished if necessary, before mounting on a glass microscope slide with clear resin (Cytoseal 60, Fisher Scientific, Pittsburg, PA, USA) and examined under transmitted light using a dissecting microscope with a digital camera connected to a computer.

Pairs of bands consisting of one highly calcified (opaque) band and one less-calcified narrow (translucent) band were identified following the description and terminology detailed in Cailliet and Goldman [19]. The birth band (BB) was distinguished as a distinct band or an angle change in the corpus calcareum [19] at a mean distance of 0.6 ± 0.05 mm (SD) from the focus (n = 220). The radius (*R*) and the distance from the focus (*F*) to the outer edge of the last (*R_n_*) and penultimate (*R_n-1_*) complete translucent bands were measured along a diagonal on the corpus calcareum on a digital image of each vertebra using the Image Pro Plus 7.0 Software (Media Cybernetics, Maryland, USA). Regressions of disc width (DW) vs. *R* were fitted to male and female data to examine whether growth of vertebral centra remained proportional to somatic growth, and an ANCOVA was used to test for differences between the two relationships.

As a preliminary training exercise, two readers (P.A. Mejía-Falla and A.F. Navia) counted the growth bands on a subsample (n = 100) both on digital images and under a microscope for discussion and interpretation. Subsequently, the two readers read vertebral sections simultaneously, but independently and without knowledge of sex or length of specimens. The process was repeated twice. Vertebral-age estimates were compared and ages that differed were re-read simultaneously by both readers; when an agreement could not be reached on an age, the sample was discarded and excluded from the age analysis [21].

### Precision and bias

Several methods were used to evaluate precision and bias of age determinations, following the recommendations of Cailliet and Goldman [19] and assuming that replicate readings were statistically independent. The methods used were: percentage agreement between readers (*PA* =  Number agreed/Number read*100) and *PA* ± 1 year and ± 2 years for all individuals and ± 1 year only for individuals grouped in 4-cm DW intervals [22]; age-bias plots of mean band count of reader 2 vs band count of reader 1 [23]; Bowker's test of symmetry [24], which determines whether differences between readers are systematic or a result of random error; and finally, the average percentage error (*APE*; [25]), which was calculated as: 
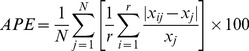
(1)where 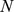 is the number of samples, *r* the number of readings, *X_ij_* is the *ith* age determination of the *jth* fish, and *X_j_* the average age calculated for the *jth* fish.

### Marginal increment

Verification of the annual periodicity of band formation was performed using marginal increment ratio analysis (*MIR*) following Natanson et al. [26] and the simplified equation of Conrath et al. [27], as:
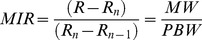
(2)where *MW* is the margin width, and *PBW* is the previous band pair width. When only one band was present, *MIR* was calculated as the proportion between the outer edge of the translucent zone and *R*. Mean *MIR* was plotted against month of capture to determine trends in band formation and a non-parametric Kruskal–Wallis one-way analysis of variance was used to test for differences in *MIR* between months; Dunn post-hoc tests were used when differences were found. *MIR* was plotted considering the number of bands of each vertebra (1–2, 3–4, ≥5 and overall sample).

### Growth curves

Eight models were fitted to the individual disc width and total length of each female and male *U. rogersi* at their estimated age at capture to estimate growth parameters. The models fitted were: von Bertalanffy with two (VBG-2) and three (VBG-3) parameters [28], Gompertz with two (GG-2) and three (GG-3) parameters [29], logistic with two (LG-2) and three (LG-3) parameters [29], and the two-phase model with five (TPG-5) and four (TPG-4) parameters [30] (Table S1). In all cases, growth parameters were estimated by fitting the model to the observed data through maximum likelihood, using SAS software (version 9, SAS Institute, Inc.).

These models involve as one of their parameters the theoretical asymptotic size (*L_∞_*). The VBG models include a relative growth rate parameter (*k_1_*, yr^−1^) and the theoretical age at zero disc width or length (*t_1_*). The GG models include the rate of exponential decrease (*k_2_*, yr^−1^) of the relative growth rate (λ) with age and a parameter *t_2_*  =  (*ln* λ–*lnk_2_*)/*k_2_*. The LG models include a relative growth rate parameter (*k_3_*, yr^−1^) and an inflection point of the sigmoidal curve (*t_3_*) [31]. The TPG models consider as additional parameters, the age at which the transition between the two phases occurs (*t_h_*, inflection point), and the maximum difference in length-at-age between the VBG and the TPG models at the point *t_h_* (*h*; Table S1). Models with two parameters require the mean size-at-birth (*L_o_*) instead of *t* values; we used a mean size-at-birth of 8.0 cm DW and 13.5 cm TL for *U. rogersi*, estimated from observed near-term embryos and smallest free-swimming individuals during this study and recorded by Mejía-Falla et al. [5].

Four data sets were built using two different size metrics and considering or not reproductive seasonality, following the recommendations of Cailliet et al. [11] and Harry et al. [7], respectively. Disc width and total length of female and male *U. rogersi* were used as size metrics. In terms of reproduction, it was assumed that the first band is formed one year after birth, irrespective of reproductive seasonality (unadjusted analysis), but the time between birth and first band formation was also considered, adjusting it to the reproductive cycle of the species (adjusted analysis). In the Colombian Pacific *U. rogersi* has three birth peaks per year (September, January and May; [5]) and the month of band formation is January (see Results below), thus the time between births and formation of the first band is 4, 12 and 8 months, respectively, with a mean of 0.67 years; in this case, the age at first band (AAFB) is adjusted to the mean value for the population.

Thus, the eight growth models were fitted to two data sets for each size metric: 1) DW (or TL)-Unadjusted and 2) DW (or TL)-Adjusted. In the unadjusted data sets for example, BB  =  0+, AAFB  =  1, age at the second band (AASB)  =  2, whereas in the adjusted data sets BB  =  0.67, AAFB  =  1.67, and AASB  =  2.67.

### Model selection

A maximum likelihood (ML) method combined with Akaike's Information Criterion (AIC) [32] was used to select the model that best fitted *U. rogersi* size-at-age data [33], [34]; additionally, the error sum of squares (SSE) and the residual mean square error (MSE) were also considered to evaluate model goodness of-fit. The model with the lowest AIC, SSE and MSE values was considered as the most probable for the data. AIC, computed as 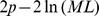, penalizes the complexity of the model, given by the number of parameters (*p*), by attaining an optimum between parsimony and accuracy [35]. AIC differences (Δ_i_  =  AIC_i_−AIC_min_) were used to rank the support of the remaining models relative to the best-fit model. Models with Δ_i_ of 0–2 had substantial support, models with Δ_i_ of 4–7 had considerably less support, and models with Δ_i_ > 10 had essentially no support [33]. The probability of choosing the true model among *k* models, termed AIC weight (*w*), was also computed from Δ_i_, as follows [33], [34]: 
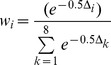
(3)


Based on multi-model inference [33], when a model had *w_i_* ≤ 90%, or more than one model had substantial support, average *L_∞_* (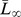) values may be estimated across those models with substantial support from the data, as well as their unconditional standard errors 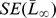, as follows:
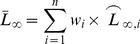
(4)

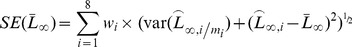
(5)where 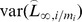
is the variance of the estimated asymptotic length according to model m_i_.

Finally, differences in growth curves between sexes for the selected model were tested using chi-square tests of likelihood ratios [36], [37].

### Longevity

Because theoretical longevities calculated from the k value obtained from growth functions are likely overestimates [38], we followed the proposal of Barnett et al. [39] to define longevity. These authors defined the upper range of longevity (ω) from maximum observed age (T_max_) and the average percentage error (APE) calculated using empirical data from the greatest 20% of age classes sampled, thus:
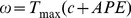
(6)where *c* is an arbitrary constant (*c*  =  1.4) to account for the likelihood that the absolute maximum age of each species was not observed in the life history study. For this study, we calculated the APE from the greatest 18% of age classes sampled (corresponding to individuals with six or more growth bands).

### Age at maturity

Median age-at-maturity (A_50_) was estimated for females and males from directly aged individuals (i.e., not back-transforming length into age) by fitting a logistic regression model to binomial maturity data (0-inmature, 1-mature) [40] using maximum likelihood. The equation used was: 
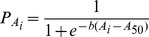
(7)where *P_Ai_* is the proportion of mature individuals at the *ith* age class and *b* is a model parameter. Differences in logistic models between sexes were tested with chi-square likelihood ratio tests [36], .

## Results

### Treatment of vertebrae and reading of bands

**Figure 1:**
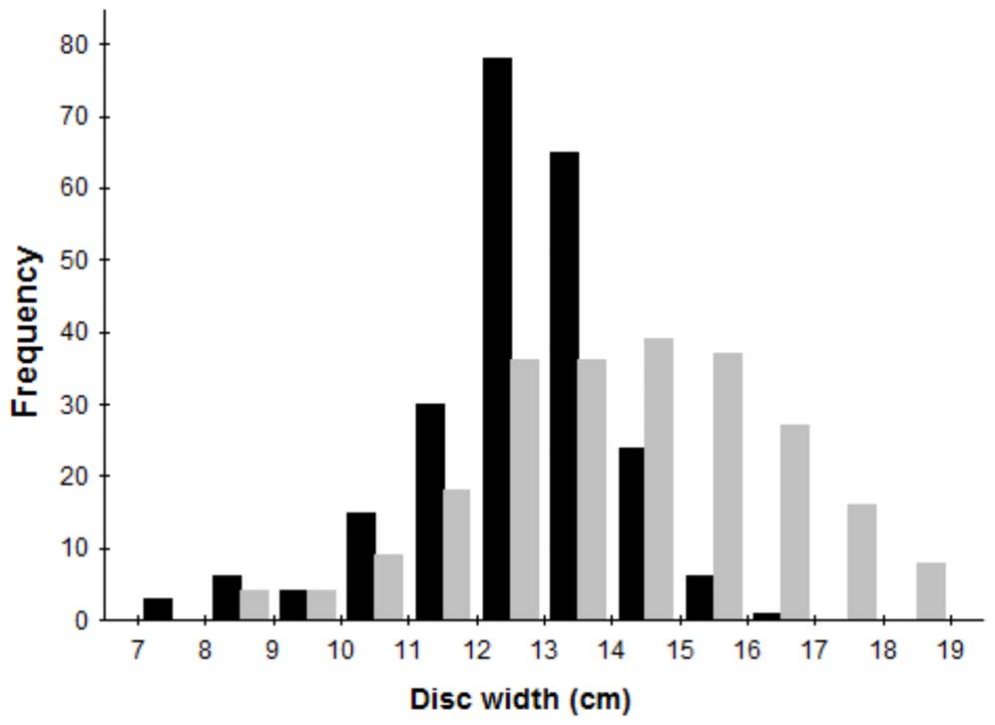
Length-frequency distributions for female (gray bars) and male (black bars) *U. rogersi* used in this study (n = 466).

A total of 503 specimens (256 male and 247 female) were initially used for the ageing study. Growth bands were distinguishable both along the intermedialia and the corpus calcareum, hence band pair counts were derived from bands on the corpus calcareum; in general, little difficulty was encountered in estimating the age of *U. rogersi*. Of the processed vertebrae, 466 were readable (92.6%), from 232 males ranging in size from 7.9 to 17.0 cm DW (mean ± SD  =  12.8 ± 1.5) and 234 females ranging from 8.2 to 18.8 cm DW (14.4 ± 2.2; Figure 1). Band pair counts of these individuals ranged between 0 and 8 in females and 0 and 6 in males.

Significant, non-linear relationships between DW and *R* (*P*  =  0.001) were found for both sexes (females: DW  =  12.3 + 18.9 * log*R*, *r^2^*  =  0.81; males: DW  =  12.3 + 17.0 * log*R*, *r^2^*  =  0.74), verifying vertebral centra as useful ageing structures. ANCOVA showed significant differences between sexes for the regression of DW vs. *R* (*F*  =  614.81, d.f.  =  244, *P*  =  0.001); therefore, the data were treated separately for each sex.

### Precision and bias

**Figure 2:**
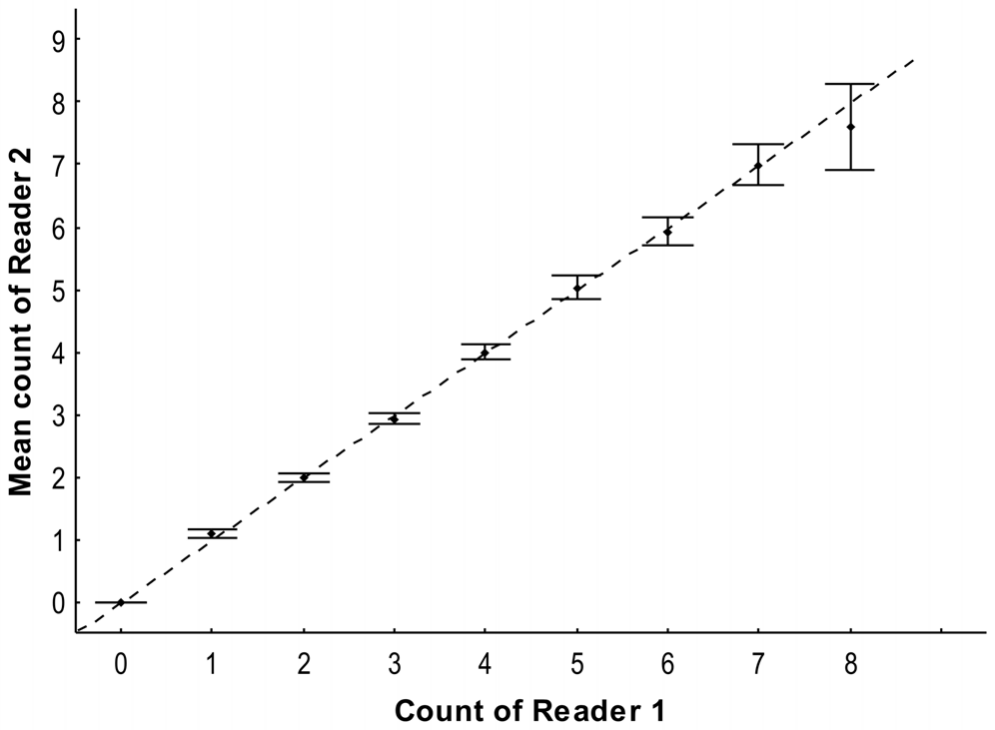
Age-bias plot of reader band pair counts. Dots with error bars are the mean counts of reader 2 (upper and lower 95% confidence limits) relative to reader 1. The diagonal line indicates a one-to-one relationship.

Age estimates agreed closely between readers. The first set of band pair counts resulted in an *APE* between readers of 3.5%, with a *PA* of 82.1%, *PA* ± 1 band of 99.3%, and *PA* ± 2 bands of 100%. When grouped by 4-cm DW intervals, agreement was reached for 95.9% and 100% ± 1 band for rays ≤ 11.4 cm DW, 83.4 and 99.4% ± 1 band for rays > 11.4 cm and ≤ 15.5 cm DW, and 68.7 and 98.8% ± 1 band for rays > 15.5 cm DW of samples initially read. The age-bias plot indicated there was no systematic bias between readers (Figure 2) and Bowker's test of symmetry indicated no systematic disagreement between readers (*χ^2^*  =  5.90, d.f  =  11, *P*  =  0.12). These precision and bias values indicated a high level of reproducibility.

### Marginal increment

**Figure 3:**
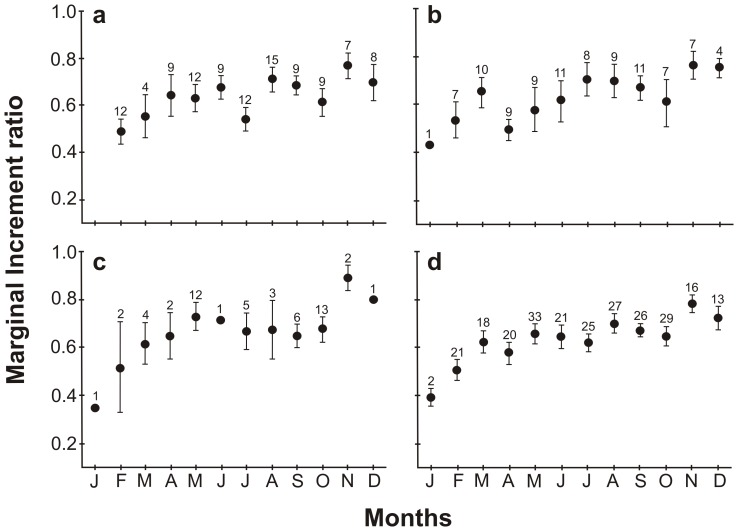
Mean marginal increment ± 1 SE by month of capture for vertebral centra of *U. rogersi* with different numbers of translucent bands. a) 1–2 bands, b) 3–4 bands, c) > 5 bands, d) overall sample. No individuals with 1–2 bands were available for January. Numbers denote sample size.

Marginal increments were significantly different among months (Kruskal-Wallis H_11,251_  =  27.2, *P*  =  0.004), with an increasing trend from January to a peak in November (Figure 3*d*). Post-hoc tests showed significant differences between February and November (*P*  =  0.003). Similar monthly trends were found on vertebral centra with 1–2, 3–4 and >5 translucent bands (Figure 3*a*–*c*), with more variability for centra with ≥ 5 bands. These results suggest that a single narrow band is formed annually on vertebral centra of *U. rogersi* during January.

### Growth curves

Based on AIC values, the majority of models with two parameters showed no empirical support (Δ*_i_* > 10) for females (Table 1) or males (Table 2; except for the two-parameter VBG model with the adjusted-DW data set). Most of those models underestimated *DW_∞_* and overestimated *k* values. In contrast, the 5-parameter TPG model had the lowest AIC and highest *w* values in data sets based on DW and TL for females, with substantial empirical support (Δ_i_ < 2) and reasonable estimates of *k* and *DW_∞_*. Values of *L_o_* were very high in all models based on TL and comparatively higher in the unadjusted than in the adjusted data sets (Table 1). Similarly in males, TPG-5 and TPG-4 had the lowest AIC values for unadjusted and adjusted data sets, respectively, based on both DW and TL (Table 2). Although the unadjusted-DW data set had the highest *w* value (92% in TPG-5), *DW_o_* was high, whereas the corresponding value obtained with the adjusted-DW data set was closest to observed size at birth. For this reason, and based on multi-model inference, an average parameter value from the two TPG models was estimated from the adjusted-DW data set (joined *w*  =  97%; Table 2).

**Table 1:** Growth model parameter estimates of female *U. rogersi* using two different values for the first growth band deposited (unadjusted and adjusting the time between months of births and band formation; see text for details), two metrics (disc width, DW, and total length, TL) and eight models (see Table S1 and methods for details).

Dataset	Model	*L∞*	*k*	*Lo*	MSE	SSE	AIC	Δ_i_	*w*
Unadjusted- DW	VBG-3	19.41	0.22	10.91	1.20	280.45	712.44	3.29	0.15
	VBG-2	16.69	0.64	8.00	2.22	519.96	854.90	145.75	0.00
	GG-3	18.79	0.29	10.95	1.21	282.39	714.05	4.90	0.07
	GG-2	16.41	0.85	8.00	2.30	537.18	862.52	153.37	0.00
	LG-3	18.39	0.37	11.00	1.22	284.45	715.75	6.60	0.03
	LG-2	17.38	0.42	11.29	1.28	298.58	725.09	15.94	0.00
	TPG-5	20.07	0.23	10.79	1.16	271.85	709.15	0.00	0.76
	TPG-4	17.29	0.68	8.00	2.07	484.53	842.38	133.24	0.00
Adjusted- DW	VBG-3	19.36	0.22	9.63	1.14	267.06	700.98	3.54	0.14
	VBG-2	17.46	0.40	8.00	1.25	292.95	720.64	23.19	0.00
	GG-3	18.74	0.30	9.80	1.15	269.55	703.16	5.71	0.05
	GG-2	17.00	0.54	8.00	1.32	307.95	732.32	34.88	0.00
	LG-3	18.34	0.37	9.94	1.16	272.08	705.35	7.90	0.02
	LG-2	17.13	0.35	11.17	1.49	349.20	761.74	64.29	0.00
	**TPG-5**	**20.08**	**0.22**	**9.40**	**1.11**	**258.59**	**697.45**	**0.00**	**0.80**
	TPG-4	17.98	0.40	8.00	1.18	275.22	710.03	12.58	0.00
Unadjusted- TL	VBG-3	35.27	0.26	19.84	5.02	1174.18	1047.51	3.20	0.15
	VBG-2	30.87	0.72	13.50	9.81	2296.55	1202.48	158.18	0.00
	GG-3	34.30	0.33	19.93	5.05	1181.73	1049.01	4.70	0.07
	GG-2	30.33	0.97	13.50	10.13	2370.03	1209.85	165.55	0.00
	LG-3	33.66	0.41	20.04	5.09	1190.00	1050.64	6.33	0.03
	LG-2	32.48	0.45	20.50	5.21	1220.01	1054.47	10.16	0.00
	TPG-5	35.63	0.29	19.57	4.87	1138.59	1044.30	0.00	0.74
	TPG-4	32.23	0.74	13.50	9.16	2143.16	1190.31	146.00	0.00
Adjusted-TL	VBG-3	35.16	0.26	17.12	4.77	1115.93	1035.60	2.61	0.19
	VBG-2	32.06	0.45	13.50	5.28	1235.00	1057.32	24.33	0.00
	GG-3	34.20	0.34	17.51	4.81	1126.36	1037.78	4.79	0.07
	GG-2	31.03	0.64	13.50	5.51	1277.87	1058.23	25.24	0.00
	LG-3	33.58	0.42	17.83	4.86	1137.03	1039.98	6.99	0.02
	LG-2	31.99	0.38	20.27	5.93	1388.21	1084.69	51.70	0.00
	TPG-5	35.60	0.28	16.52	4.62	1080.23	1032.99	0.00	0.72
	TPG-4	33.25	0.45	13.50	4.92	1150.49	1044.74	11.75	0.00

*L_∞_* is the theoretical asymptotic size, *k_i_* is the coefficient of growth, *L_o_* is the birth size, MSE is the mean square error of the residuals, SSE is the error sum of squares, AIC is Akaike's Information Criterion, Δ*_i_* is the Akaike difference, and *w* is the AIC weight.

**Table 2:** Growth model parameter estimates of male *U. rogersi* using two different values for the first growth band deposited (unadjusted and adjusting the time between months of births and band formation; see text for details), two metrics (disc width, DW, and total length, TL) and eight models (see Table S1 and methods for details).

Dataset	Model	*L∞*	*k*	*Lo*	MSE	RSS	AIC	Δ_i_	*w*
Unadjusted- DW	VBG-3	15.11	0.56	10.49	0.82	191.08	619.37	5.91	0.05
	VBG-2	14.22	1.28	8.00	1.65	383.08	778.74	165.28	0.00
	GG-3	14.97	0.65	10.51	0.83	192.35	620.90	7.44	0.02
	GG-2	14.12	1.55	8.00	1.66	386.04	780.52	167.07	0.00
	LG-3	14.86	0.75	10.53	0.83	193.66	622.48	9.02	0.01
	LG-2	15.30	0.69	10.36	0.85	197.66	625.22	11.76	0.00
	**TPG-5**	**15.40**	**0.69**	**10.38**	**0.79**	**183.09**	**613.46**	**0.00**	**0.92**
	TPG-4	15.09	1.27	8.00	1.56	362.58	769.98	156.52	0.00
Adjusted- DW	VBG-3	15.05	0.56	8.57	0.76	177.17	601.84	8.99	0.01
	VBG-2	14.80	0.66	8.00	0.77	178.23	601.22	8.37	0.01
	GG-3	14.90	0.67	8.69	0.77	178.94	604.14	11.30	0.00
	GG-2	14.56	0.84	8.00	0.78	181.68	605.67	12.82	0.00
	LG-3	14.79	0.77	8.86	0.78	180.78	606.52	13.67	0.00
	LG-2	14.85	0.55	10.16	0.87	202.82	631.20	38.35	0.00
	**TPG-5**	**15.43**	**0.66**	**7.98**	**0.73**	**169.04**	**594.94**	**2.09**	**0.25**
	**TPG-4**	**15.47**	**0.64**	**8.00**	**0.73**	**168.98**	**592.85**	**0.00**	**0.72**
Unadjusted- TL	VBG-3	28.01	0.63	19.71	5.86	1358.44	1074.41	2.91	0.16
	VBG-2	26.48	1.56	13.50	11.97	2777.90	1238.38	166.88	0.00
	GG-3	27.78	0.74	19.74	5.88	1363.37	1075.25	3.75	0.10
	GG-2	26.32	1.88	13.50	12.01	2787.06	1239.14	167.64	0.00
	LG-3	27.59	0.85	19.76	5.90	1368.42	1076.11	4.61	0.07
	LG-2	31.69	0.44	20.13	6.14	1424.32	1083.40	11.90	0.00
	TPG-5	28.83	0.78	19.52	5.68	1318.56	1071.50	0.00	0.67
	TPG-4	28.34	1.49	13.50	11.60	2691.79	1235.07	163.57	0.00
Adjusted-TL	VBG-3	27.50	0.74	14.75	5.44	1263.04	1057.52	7.00	0.02
	VBG-2	27.06	0.87	13.50	5.47	1268.72	1056.56	6.04	0.03
	GG-3	27.33	0.85	15.37	5.49	1274.61	1059.64	9.12	0.01
	GG-2	26.65	1.12	13.50	5.56	1290.87	1060.58	10.06	0.00
	LG-3	27.20	0.95	15.84	5.54	1285.10	1061.54	11.02	0.00
	LG-2	27.63	0.67	18.28	5.79	1342.87	1069.74	19.22	0.00
	TPG-5	28.53	0.83	13.66	5.24	1214.76	1052.48	1.96	0.25
	TPG-4	28.49	0.84	13.50	5.24	1214.97	1050.52	0.00	0.68

*L_∞_* is the theoretical asymptotic size, *k_i_* is the coefficient of growth, *L_o_* is the birth size, MSE is the mean square error of the residuals, SSE is the error sum of squares, AIC is Akaike's InformationCriterion, Δ*_i_* is the Akaike difference, and *w* is the AIC weight.

Based on statistical results (MSE, SSE, AIC and *w* values) and biological interpretation (*k*, *L_∞_* and *L_o_* values), the 5-parameter TPG based on the adjusted-DW data set was deemed to provide the best description of growth for females (*w*  =  80%) with *DW_∞_*  =  20.1 cm (SE  =  0.10) and *k*  =  0.22 yr^−1^ (SE  =  0.05; Table 1). For males, the asymptotic disc width (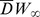) estimated through multi-model inference from TPG-5 and TPG-4 was 15.5 cm (SE  =  0.63) and *k* was 0.65 yr ^–1^ (SE  =  0.001). To allow comparison with the TPG-5 model for females, *t_o_*, *t_h_, h* and *DW_o_* were estimated by fitting this function to the adjusted-DW data and fixing 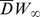; these values were, −1.14, 3.2 years, 0.25 and 7.99 cm, respectively.

**Figure 4:**
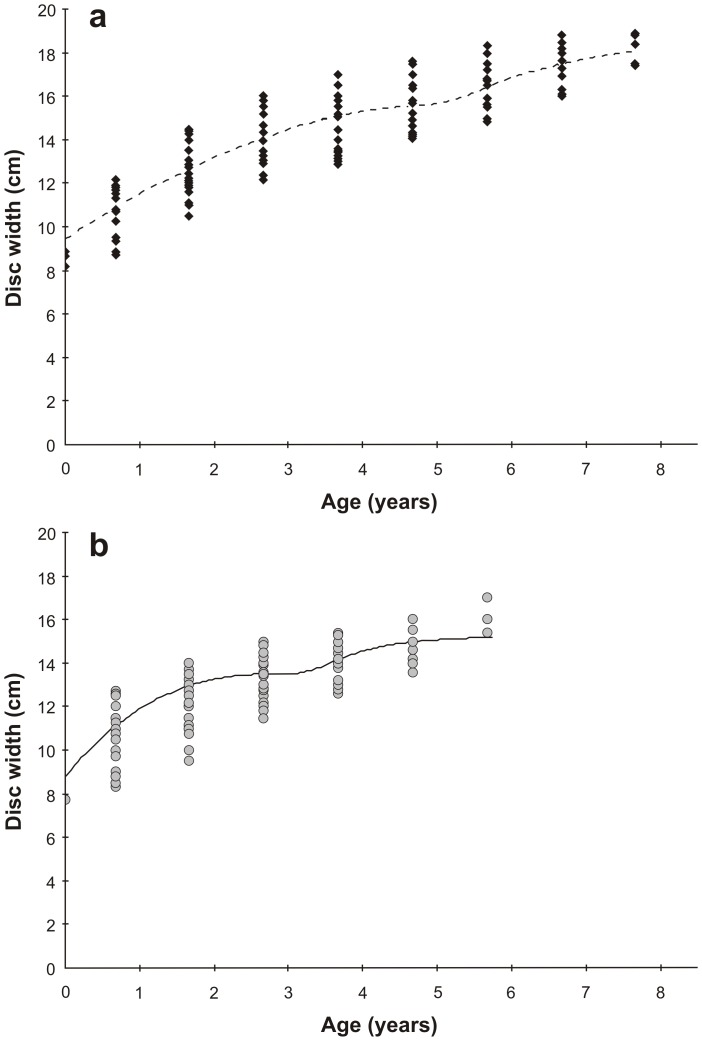
Growth curves for female (a) and male (b) *U. rogersi* using the single (five parameter) and average (5 and 4 parameter) Two-Phase Growth Model, respectively, from the adjusted-*DW* data set.

Two-phase growth curves were significantly different between sexes (likelihood ratio χ^2^  =  36.04, d.f  =  5, *P* < 0.0001). Female *U. rogersi* had a lower growth coefficient than males and a larger asymptotic size (Figure 4*a,b*). The model estimated well the size-at-birth for males (8.0 cm DW), but overestimated that of females (9.4 cm DW), although less than the other models (except those with two parameters in which this value was fixed). For females, the change in growth rate (*t_h_*, inflection point of the curve) occurred later (5.1 ± 0.3 years) than in males (3.2 ± 0.2 years), corresponding to 15.7 and 13.6 cm DW, respectively (Figure 4*a,b*). Sizes corresponding to maximum observed ages also differed between sexes, with the oldest aged female being near 8 years and 19 cm DW, and the oldest male, near 6 years and 17 cm DW.

### Longevity

The APE value of the greatest 18% of age classes sampled was 3.01%. Thus, from this value and based on the Barnett et al. [39] equation, the longevity for *U. rogersi* was calculated at 11 years for females and 8 years for males.

### Maturity

**Figure 5:**
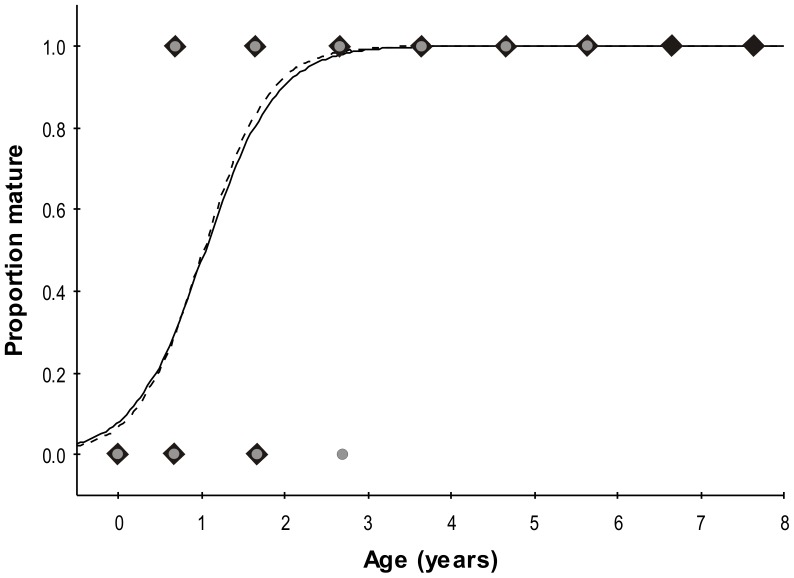
Maturity ogives for age fitted to binomial data for female (black diamonds, dotted line) and male (white circles, solid line) *U. rogersi*.

Estimates of A_50_ were low and equal for females and males (1.0 year; SE  =  0.1; 95% CI  =  0.9–1.2; Figure 5). Likelihood ratio tests showed no differences between sexes in the relation between maturity and age (logistic curve: χ^2^  =  0.54, d.f  =  2, *P*  =  0.765; A_50_: χ^2^  =  0.18, d.f  =  1, *P*  =  0.67; b: χ^2^  =  0.46, d.f  =  1, *P*  =  0.50).

## Discussion

Age and growth studies of batoids have been conducted mainly on skates and to a lesser extent on stingrays [19]. These studies have been carried out in only five of 24 species of stingarees (family Urolophidae) and one of 16 species of American round stingrays (family Urotrygonidae; Table 3). The present is the first age and growth study for the genus *Urotrygon*, and its results are in contrast with the generalization that elasmobranchs are slow-growing, long-lived species. This finding has considerable implications for the population assessment and management of *U. rogersi* in particular and suggests reconsidering the previously mentioned generalization about elasmobranch growth.

**Table 3:** Comparison of growth parameter estimates of related species of the families Urolophidae and Urotrygonidae obtained with the von Bertalanffy growth model.

Species	Study area (Ocean, country)	Sex	*DW_∞_* (cm)	*k* (yr^−1^)	Maximum age (year)	Maximum DW (cm)	Maturity age (year)	n	References
*Urotrygon rogersi*	TE Pacific, Colombia	F	19.4	0.22	8.0	19.9	1.0	234	This study
		M	14.8	0.63	6.0	17.0	1.0	232	
*Urobatis halleri*	NE Pacific, USA	F	22.5	0.15	14.0	21.3	3.8	96	[9]
		M	28.6	0.09	14.0	23.9	3.8	84	
*Urobatis halleri*	NE Pacific, USA	F	31.0	–	8.0	–	2.6	96	[55]
		M	25.0	–	8.0	–	2.6	84	
*Urolophus lobatus*	SE Indian, Australia	F	24.1	0.26	14.0	27.7	–	330	[42]
		M	20.3	0.36	12.0	23.7	–	437	
*Urolophus paucimaculatus*	SE Indian, Australia	F	26.1	0.27	14.0	27.2	5.0	113	[41]
		M	24.3	0.36	11.0	25.6	3.0	99	
*Urolophus paucimaculatus*	SE Indian, Australia	F	57.3*	0.21	10.0	56.0*	3.0	113	[54]
		M	42.8*	0.45	8.0	44.0*	–	99	
*Trygonoptera personata*	SE Indian, Australia	F	30.3	0.14	16.0	31.1	4.0	352	[43]
		M	26.9	0.20	14.0	26.9	4.0	303	
*Trygonoptera mucosa*	SE Indian, Australia	F	30.8	0.24	17.0	36.9	5.0	324	[43]
		M	26.1	0.49	14.0	28.3	2.0	400	

TE: Tropical Eastern; NE: North Eastern; SE: South Eastern. F: female, M: male. * Values are total length.

Despite their small size, vertebrae are good structures to evaluate and estimate the age and growth of *U. rogersi*, as used for other species of Urotrygonidae and Urolophidae [9], [41]–[43]. Based on our results and those of other studies of urolophids and urotrygonids, we suggest that the technique used in this study (0.4 mm sections and unstained vertebrae) is appropriate as a starting point for other species of *Urotrygon* or *Urobatis*, but recommend testing thicknesses between 0.3 and 0.5 mm.

Formation of one annual band pair has also been verified through marginal increment analysis in closely related species from other geographical areas, such as *U. lobatus*, *Trygonoptera personata* and *T. mucosa* from the South West coast of Australia [37], [38] and *U. halleri* from the West coast of the USA [35], [44]. Although marginal increment analysis has been considered as a validation method [45], in this study we use it as a verification method as proposed by Cailliet and Goldman [19] to verify the frequency of band deposition. However, marginal increment analysis has proven problematic owing to technical difficulties related to resolving the margins of growth bands [45] and because the number of deposited bands and time of deposition may vary with age. In this regard, verification of the periodicity of band deposition should be conducted by age class [45]–[47], as in the present study. Verification of annual band formation in *U. rogersi* was likely facilitated by the fast growth and short lifespan of the species, and an adequate sample size.

### Growth curves

In viviparous species, and particularly in *U. rogersi*, disc width has been associated with reproductive aspects. For instance, disc width is thought to limit fecundity and embryo maximum size [5], [48]. Thus, the use of disc width is more sensible than that of total length from a biological standpoint, and is recommended for age and growth studies of myliobatiform batoids (i.e. the families Dasyatidae, Urotrygonidae, Urolophidae, Gymnuridae, Myliobatidae, and Potamotrygonidae).

Although no analyses of vertebrae of near-term embryos were included in this study, the smallest free-living *U. rogersi* (an 8.2 cm DW female captured in August, and two 7.9 cm DW males captured in August and January) did not have any evidence of bands, indicating that the first band is formed after birth. This supports our use of an adjusted data set, especially for tropical species with multiannual reproductive cycles or even those with no seasonal reproductive cycles. Age data of aseasonal species with reproductive events throughout the year can be adjusted by averaging the time between each month of possible birth and the month of band formation [7], resulting in an adjustment of 0.54 years in our case.

Two-parameter models incorporating estimated size at birth were not deemed to be appropriate descriptors of DW-at age data for *U. rogersi* because *DW_∞_* underestimated observed maximum DW values and appeared to overestimate *k* values in both females and males, making them a biologically unreasonable choice in most cases. In contrast, models with at least three parameters generally described observed size-at-age data well. Thorson and Simpfendorfer [49] used simulation to show that two-parameter models were only useful for small samples sizes (<100) after which three- or four- parameter models performed better. However, these authors also suggested that sample sizes of 200 are required to consistently achieve good accuracy for growth parameters, which supports the results of this study for both sexes (females, n = 234; males, n = 232).

Moreau [50], Wang and Milton [51] and Katsanevakis and Maravelias [31] proposed that the choice of the best growth model and the interpretation of estimated parameters should be subjective and, in some cases, based on the decision of the researcher, founded on experience with the species and previous studies. In this study, the TPG-5 model based on unadjusted-DW was not selected as the best model to describe male growth despite having the highest empirical support and AIC weight because the estimated *DW_o_* was not reliable. Instead, we opted to use multi-model inference by averaging the TPG-4 and TPG-5 models to produce more robust parameter estimates (with unconditional standard errors) for males and allow comparison with the growth model for females [17], [31].

For batoids, it has been proposed that the Gompertz model describes growth better than other models, especially in rays that continue to grow in weight but not greatly in length [19] and those with viviparous reproduction [52]–[54]. Although *U. rogersi* reaches a large adult weight in relative to its juvenile weight and is a viviparous species, decision criteria showed that the Gompertz model was not appropriate in this case, performing worse than the TPG or even the three-parameter VBG models.

The suitability of the TPG model, which incorporates the influences of changes in growth trajectories [32], has been evaluated for some elasmobranch species. Araya and Cubillos [35] associated the change in growth with the onset of maturity. In female and male *U. rogersi*, the inflection points (*t_h_*  =  5 and ≈ 3 years, respectively) are higher than the A_50_ values (one year in both cases); therefore we suggest that the onset of maturity does not influence this change. However, given the small size at which maturity is reached, it is necessary for individuals to invest energy in growth in parallel to reproduction to achieve greater reproductive success in their lifetime. Females may continue growing to a size or age at which fecundity is highest (3 embryos), i.e., 16.0 cm DW [5], which coincides with the inflection point at ≈ 5 years in the TPGM (Figure 3). Thus, this inflection point is associated more with increased reproductive output (i.e., higher fecundity) than with the onset of maturity.

It is possible that other events or discontinuities in development, such as changes in habitat or behavior, may cause changes in growth trajectories [29]. Since no segregation by sex or size is apparent in the area where individuals of all ages and sizes are found throughout the year [5], no changes in habitat are suggested. Changes in feeding habits with size or age can generate changes in behavior; ontogenetic changes in diet were detected in *U. rogersi* from individuals < 20 cm total length (< 11.5 cm DW, ca. 1 year old) compared to larger ones, with a shift from polychaetes to shrimps [4]. This size or age is closer to that corresponding to the onset of maturity than to the inflection point in the growth curve.

As the present study is the first to provide age and growth estimates for *U. rogersi*, no comparisons of growth parameters with other populations within the Eastern Tropical Pacific region are possible. In addition, as this is the first age and growth study for a batoid species on the Colombian Pacific Ocean, no comparisons with sympatric congeners are possible. Comparisons with stingarees or round stingrays from Southwest Australia (genera *Urolophus* and *Trygonoptera*) and American round stingrays of the west coast of the USA (genus *Urobatis*), based on available von Bertalanffy growth model parameters, suggest that female *U. rogersi* complete their growth at a similar rate (*k* value) than *U. lobatus*, *U. paucimaculatus* [54] and *T. mucosa* and reach a maximum age similar to that of *U. halleri* ([55], Table 3). Male *U. rogersi* appear to complete their growth at a higher rate and reach a lower maximum age and size than those other species (Table 3). Females of these species have greater *DW_∞_* and lower *k* values than males, except for *U. halleri* [9], although this discrepancy may have been caused by the paucity of larger females sampled in that study. Thus, larger sizes, slower growth, and longer lifespan in females than males seems to be a general pattern for the stingarees or round stingrays (families Urolophidae and Urotrygonidae) and the result of an ancestral trait mediated by intrinsic (i.e., advantages in reproduction) rather than extrinsic environmental factors.

Interpretation of growth coefficients for a species is influenced by sample sizes, size ranges, ageing methodology, validation of band formation periodicity, and model fitting techniques [19]. Thus, although growth coefficients are not directly comparable among models, they may still provide a practical but generalized characterization of fundamental life history traits that may be linked to longevity, fecundity, and size or age at maturity [56], [57]. According to Holden [58], growth coefficients for batoid elasmobranchs (based on linear relationships or the VBG model) range from 0.1 to 0.3. Based on this, females and males of some species fall within the slow-growing end of this range (i.e., *U. halleri*, *T. personata*), whereas others, notably male *U. rogersi*, fall outside, toward the fast-growing end of this range.

Beukema [59] suggested that maximum age directly estimated from a population may not provide an adequate measure of species longevity. However, theoretical estimates of longevity derived from the growth coefficient alone are also influenced by the choice of growth model and therefore could be a mathematical artifact rather than reflect biological reality, for which reason we did not use them in this study. Particularly for female *U. rogersi*, the theoretical longevity, defined as the age at which 95% or 99% of *DW_∞_* is reached, would be 15 or 22 years, respectively, which is very high, as much as three times the corresponding values for males (5 or 7 years). Considering the sample size, age at maturity of the species (about 1 year), and the definition of theoretical longevity, these theroretical values of longevity for females appear to be overestimates, as noted by others authors [38], [60]. Thus, we believe the longevity estimates obtained by using the equation proposed by Barnett et al. [39] based on the APE value are more realistic for this species because they approximate the maximum observed age much better.

### Age at maturity

Growth and reproduction should not be separated as different aspects of life history because they are inter-related. The onset of maturity involves a reduction in the energy allocated to growth because more energy is shifted towards reproduction [61]. Moreover, it has been suggested that a better strategy is to stop growing once maturity is reached and to invest all available energy into reproduction thereafter (“bang-bang strategy”) than to continue growing while reproducing (“intermediate strategy”; [62], [63]). However, male and female *U. rogersi* seem to follow the latter strategy, wherein growth just slows down two (males) or four (females) years after the onset of maturity (when fecundity can be higher in females), and then speeds up again. Therefore growth and reproduction co-occur and individuals simultaneously invest energy in both processes.

Despite differences in maximum length and theoretical asymptotic size between male and female *U. rogersi*, both sexes reach maturity at similar sizes [5] and ages. The same occurs in *U. halleri* [55], *T. personata* [43], and *T. imitata* [64], but not in *U. lobatus* [42] or *T. mucosa* [43], in which females reach maturity at larger sizes and older ages than males. The very rapid onset of maturity in *U. rogersi* (< 1 year) is to our knowledge the earliest for any batoid species; only male *T. mucosa* reaches maturity at a young age of 2 years [43]. This rapid onset of maturity was also found in the Australian sharpnose shark *Rhizoprionodon taylori*, which matures in one year and is also a tropical species [65].

The growth patterns found in this study indicate that *U. rogersi* is a relatively short-lived, fast-growing species in which females and males reach 58% and 70% of maximum disc width, respectively, in the first year of life. This, coupled with reproductive aspects such as short gestation period, a triannual reproductive cycle, and low fecundity but with large pups [5], indicates that this species could be more resilient to exploitation than other elasmobranchs for any given level of fishing pressure. However, demographic or other population assessment models are needed to characterize the vulnerability of the studied population to fishing or other stressors.

## Supporting Information

Table S1 **Equations of the growth models used in the study.**
(DOCX)
