# Supplementary material for: Age and Growth of the Round Stingray Urotrygon rogersi, a Particularly Fast-Growing and Short-Lived Elasmobranch
Source: PLoS One. 2014 Apr 28;9(4):e96077. doi: 10.1371/journal.pone.0096077 (PMC4002482; doi:10.1371/journal.pone.0096077)
Supplement: Table S1 — Equations of the growth models used in the study. (DOCX) [file pone.0096077.s001.docx]

**Table S1.** Equations of the growth models used in the study.

| Growth model | Abbreviation | Equation |
| --- | --- | --- |
| von-Bertalanffy (3 parameters) | VBG-3 | $L_{t}=L_{\infty}\times\left(1-e^{\left(-k_{1}\left(t-t_{1}\right)\right)}\right)$ |
| von-Bertalanffy (2 parameters) | VBG-2 | $L_{t}=L_{\infty}-\left(L_{\infty}-L_{o}\right)\times e^{\left(-k_{1}t\right)}$ |
| Gompertz (3 parameters) | GG-3 | $L_{t}=L_{\infty}\times e^{\left(-e^{\left(-k_{2}\left(t-t2\right)\right)}\right)}$ |
| Gompertz (2 parameters) | GG-2 | $L_{t}=L_{o}\times\left(e^{\left(LN\left(\frac{L_{\infty}}{L_{o}}\right)\times(1-e^{(-k_{2}t)})\right)}\right)$ |
| Logistic (3 parameters) | LG-3 | $L_{t}=\frac{L_{\infty}}{(1+e^{(-k_{3}(t-t_{3}))})}$ |
| Logictic (2 parameters) | LG-2 | $L_{t}=L_{\infty}\times\left(1+\left(\frac{L_{\infty}-L_{0}}{L_{\infty}}\right)\times(e^{-k_{3}t})\right)^{-1}$ |
| Two-phase (5 parameters) | TPG-5 | $L_{t}=L_{\infty}\times\left(1-e^{\left(-k_{1}\left(t-t_{1}\right)\times\left(1-\left(\frac{h}{\left(t-t_{h}\right)^{2}+1}\right)\right)\right)}\right)$ |
| Two-phase (4 parameters) | TPG-4 | $L_{t}=L_{\infty}-(L_{\infty}-L_{o})\times\left(e^{\left(-k_{1}\left(t\right)\times\left(1-\left(\frac{h}{\left(t-t_{h}\right)^{2}+1}\right)\right)\right)}\right)$ |

*L_∞_* is the theoretical asymptotic size, representing the average size-at-age that individuals in a stock would attain if they grew indefinitely. *k_1_* (yr^-1^) is the relative growth rate, which is a curvature parameter determining the rate at which a fish reaches the asymptotic size-at-age and which decreases linearly with size, and *t_1_* is the theoretical age at zero disc width or length, which is a position parameter defining the initial condition on the time axis when mean disc width or total length-at-age is zero. *k_2_* (yr^-1^) is the rate of exponential decrease of the relative growth rate (*_λ_*) with age and *t_2_* is a parameter corresponding to (*ln_λ_ – lnk_2_*)/*k_2_*. *k_3_* (yr^-1^) is the relative growth rate and *t_3_* is the inflection point of the sigmoidal curve. *t_h_* is the age at which the transition between the two phases occurs (inflection point), and *h* is the maximum difference in length-at-age between the VBG and the TPG models at the point *t_h_* . *L_o_* is the mean size-at-birth (8.0 cm DW and 13.5 cm TL).
